# X-ViTCNN: A Novel Network-Level Fusion of Transfer Learning and Customized Vision Transformer for Multi-Stage Alzheimer’s Disease Prediction Using MRI Scans

**DOI:** 10.3390/diagnostics16060835

**Published:** 2026-03-11

**Authors:** Armughan Ali, Hooria Shahbaz, Shahid Mohammad Ganie, Manahil Mohammed Alfuraydan

**Affiliations:** 1 Department of Electrical Engineering, Wah Engineering College, University of Wah, Wah-Cantt 47040, Pakistan; armughan.ali@wecuw.edu.pk; 2 Department of Computer Science, HITEC University, Taxila 47080, Pakistan; hooria@zerothgen.com; 3 ZerothGen, Hasan Abdal 43730, Pakistan; 4 Department of Health Information Management and Technology, College of Applied Medical Sciences, King Faisal University, Al-Ahsa 31982, Saudi Arabia; mmalfridan@kfu.edu.sa

**Keywords:** Alzheimer’s disease, MRI scans, image preprocessing, network-level fusion, transfer learning, vision transformer, explainable AI

## Abstract

**Background/Objectives:** Alzheimer’s disease (AD), the most prevalent form of dementia, is characterized by an overall decline in cognitive functioning and represents a major public health crisis. It remains critical to be able to accurately and quickly diagnose patients with AD; however, recent deep learning approaches using MRI data do not provide sample generalization, have high computational requirements, and offer little interpretability. **Methods:** In this study, we present a new framework called eXplorative ViT-CNN (X-ViTCNN) that combines a customized Vision Transformer model with two previously trained CNNs (DenseNet201 and MobileNetV2). With our proposed preprocessing approach using contrast-enhanced preprocessing to highlight neuroanatomical features as well as Bayesian Optimization to tune hyperparameters, we fuse local structural features originating from the CNNs with global representations from the transformer and feed the final result to fully connected dense layers for multi-stage classification. We also use Grad-CAM visualizations to provide insight into how our model arrived at its classification. **Results:** Experiments conducted on ADNI and OASIS datasets demonstrate the superiority of X-ViTCNN, achieving accuracies of 97.98% and 94.52%, respectively. The model outperformed individual baselines and other pre-trained architectures, showing balanced sensitivity and specificity across all AD stages. **Conclusions:** The proposed X-ViTCNN framework is a powerful, interpretable method for predicting the development of multi-stage Alzheimer’s disease using MRI scans. The combination of complementary feature learning, automatic hyperparameter optimization and interpretability through visualization make it an excellent potential tool for clinicians to support their decision making in the early diagnosis and ongoing monitoring of persons with Alzheimer’s disease.

## 1. Introduction

**Figure 1:**
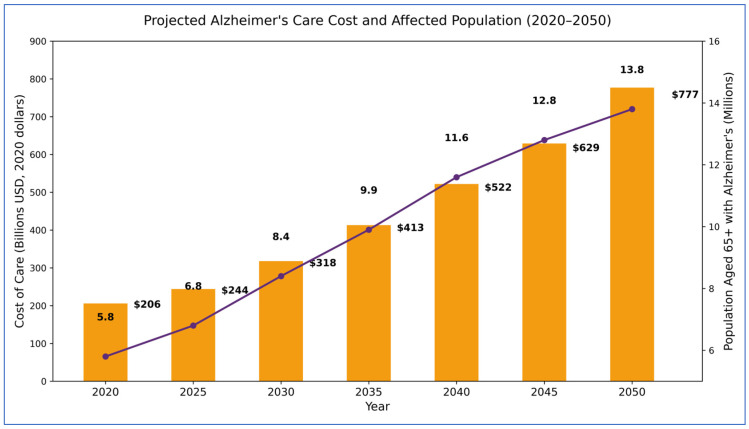
Alzheimer’s disease prevalence and costs to Medicare and Medicaid.

Alzheimer’s disease (AD), the most common type of dementia worldwide, reduces a person’s ability to carry out their daily routine and is characterized as having cognitive impairment [1,2]. The deterioration of the body’s brain cells occurs gradually and causes an individual to eventually experience problems with short-term memory and behavior. Most individuals aged 60 years old or older will experience brain cell loss through the degenerative process; however, there are a few individuals in their 40s and 50s that are currently diagnosed with AD. The Centers for Disease Control and Prevention (CDC) recently estimated that approximately five million individuals in the United States have AD, and the number of individuals diagnosed with AD is projected to increase by approximately three times before the year 2050 [3]. The resources state that, by the year 2050, approximately one out of every 85 individuals will have been diagnosed with AD [4]. All of the statistics shown in Figure 1 were collected with a focus on the cost-effectiveness of caring for the US population with AD and point to the importance of an early diagnosis of AD as there is currently no medical cure. An important factor contributing to the lack of treatment options is the utilization of machine learning (ML) and deep learning (DL) for the automated diagnosis of Alzheimer’s disease (AD) using MRI images. Previous studies indicate that there are situations where ML-based predictive models are not adequately predictive of AD [5]. Among the ML statistical models being used, Support Vector Machines (SVMs) are generally regarded as one of the more successful methods for identifying patients with AD. Alternative ML-based models such as convolutional neural networks (CNNs) and Sparse Autoencoders are at least as effective if not better than SVMs [6].

Despite notable progress in research, a specific treatment for Alzheimer’s disease (AD) remains elusive. Early detection is vital for understanding the progression of AD and reducing its effects on patients’ well-being. As illustrated in Figure 1, the number of Americans aged 65 and above with AD is expected to soar from 5.8 million in 2020 to 13.8 million by 2050, with care costs escalating from $206 billion to $777 billion (in 2020 dollars), highlighting the increasing health and economic challenges. The development of neuroimaging methods, especially MRI, has made early and precise diagnosis more achievable due to MRI’s superior contrast, resolution, and availability [7]. Several automated methods have been proposed for detecting and classifying AD by leveraging identified traits; however, many approaches often focus only on regions of interest (ROIs) or volumes of interest (VoIs), which may overlook critical features [8,9,10]. Distinctive attributes have also been extracted from different regions of the hippocampus [11,12,13] and gray matter voxels [2], further enhancing early detection. Despite these advances, most existing studies have concentrated on binary classification, indicating only the presence or absence of AD, rather than capturing its progressive stages.

Recent research demonstrated the utilization of current machine learning models, mainly CNNs, to support MRI-derived classification of cognitive state using both 3-D and 4-D modalities [14]. MRI is considered to be a valuable tool for diagnosing AD at an earlier stage because its resolution is high and it is non-invasive. The anatomical characteristics of the brain are revealed by the structure of the brain, so researchers can see and measure neuroanatomical properties related to the symptoms of AD as the disease progresses, such as a decrease in volume of the hippocampus or a decrease in the thickness of the cerebral cortex. Classic machine learning methodologies (e.g., SVMs, GMMs), while previously applied to classification problems, mainly used both advanced methodologies to extract features from MRI images and therefore were not very effective in allowing the detailed spatial heterogeneity of the brain itself. Although the findings are encouraging, these models have not been fully integrated into clinical practice due to many factors. DL algorithms require external validation since they are often taught and evaluated on a single cohort [15]. In other words, DL algorithms accurately classify various diseases but do not explain the diagnostic conclusions or identify the input characteristics linked to the output predictions. Finally, due to the unpredictable onset and variability of symptoms in AD, a comprehensive-level diagnosis of the disease remains unclear. Furthermore, the existing DL approaches train deep architectures from the beginning; however, this approach has some limitations [16,17], such as requiring more labeled data for the training, substantial computer resources, and the initialization of random hyperparameters.

Many researchers have made significant efforts to advance and refine the transfer learning model for classifying and detecting medical images. This model has been effectively utilized in various medical applications, such as distinguishing tumor cells from healthy ones [18], the identification of COVID-19, and the classification of tumors into a relevant category [19]. Despite its classification nature, transfer learning is considered computationally heavy and requires improvement in terms of computational resources. Many scientists have introduced various techniques to resolve issues, such as compressing models using prune and quantizing, designing less weighted models by controlling parameters, which can lower computational resources. However, this technique reduces the model’s accuracy and precision, which remains a potential area for further advancement. Moreover, the continuous improvement in classification techniques, such as transfer learning, is considered a problem-solving approach, and its variations have led to a significant increase in network parameters, combining various trained models. However, increasing parameters helps improve evaluation metrics. Furthermore, feature extraction techniques help to provide only relevant parameters for the model classification [20].

This study presents a novel X-ViTCNN fused model that integrates transfer learning and a customized Vision Transformer to improve Alzheimer’s disease prediction. The contributions address limitations in prior works by enhancing feature learning, optimizing hyperparameters, and ensuring interpretability. Our major contributions to this work are as follows:

**1.**  **Preprocessing Pipeline:** A max-color-based contrast enhancement technique is applied to highlight critical neuroanatomical structures. This preprocessing step significantly improves the discriminative power of the model by making subtle disease-relevant features more prominent.
**2.**  **Novel Network-Level Fusion Architecture:** The proposed X-ViTCNN model, a unique network-level fusion framework that integrates two pre-trained CNN models (DenseNet201 and MobileNetV2) with a customized Vision Transformer, enables the complementary learning of local and global features from MRI scans.
**3.**  **Customized Vision Transformer:** A lightweight ViT is designed and optimized for brain MRI analysis, addressing the challenge of limited annotated data while effectively capturing long-range dependencies.
**4.**  **Bayesian Optimization:** Bayesian Optimization is employed to automate hyperparameter selection, ensuring improved generalization, reproducibility, and computational efficiency compared to manual tuning.
**5.**  **Comprehensive Evaluation:** The framework is rigorously validated on two widely used benchmark datasets (ADNI and OASIS), demonstrating superior performance in terms of accuracy, precision, recall, specificity, and F1-score compared to individual networks and existing state-of-the-art methods.
**6.**  **Model Interpretability:** The proposed X-ViTCNN architecture is interpreted using Grad-CAM visualization, ensuring transparency in predictions by highlighting the brain regions most relevant to classification. This enhances the clinical relevance and trustworthiness of the framework.

The remainder of this paper is organized as follows. Section 2 reviews the related work on MRI-based Alzheimer’s disease detection and recent advances in deep learning and transformer-based architectures. Section 3 describes the materials and methods, including the datasets, preprocessing techniques, the proposed X-ViTCNN framework, and model training procedures. Section 4 presents the experimental results, ablation studies, statistical analysis, and discussion of clinical implications. Finally, Section 5 concludes the paper and outlines potential directions for future research. 

## 2. Related Work

Over the last decade, researchers in computer vision have developed numerous advanced techniques for the classification of AD. Some studies have concentrated on conventional methods, while a few have utilized deep learning frameworks for identifying and categorizing AD using MRI and PET images.

For example, Q Chen et al. [21] presented a framework for the diagnosis of AD using 3D MRI imaging techniques. For training and feature extraction processes, LongFormer, a hybrid 3D CNN and transformer, was developed. The authors trained and evaluated models on ADNI, OASIS, and AIBL datasets and achieved accuracies of 93.43%, 82.35%, and 85.77%, which gave a better performance than earlier techniques. The constraint of this work is that it only focuses on binary classification and cannot classify other classes of AD. S Liu et al. [22] introduced an unsupervised technique to predict pMCI from AD using non-imaging data named novelty detection (ND). In ND, they used the Gaussian mixture model (GMM), isolation forest (IF), k-means, extreme learning machine (ELM), and k-nearest neighbors (kNN). They used ADNI1, ADNIGO, ADNI2, and ADNI3 datasets for the experiment and achieved an accuracy of 72.70% for KNN, 71.79% for GMM, and 72.76% for ELM. The limitation of this research was that it cannot predict pMCI automatically with ND algorithms. RA Hazarika et al. [23] presented a DNN model for AD classification using MRI scans. They designed a hybrid model by incorporating LeNet and AlexNet, which generates fewer convolutional parameters. The proposed method was applied to the ADNI dataset and produced a 93.58% accuracy rate. Marwa et al. [24] developed an AD classification technique using 2D T1-weighted MR brain images. The presented CNN model was fast and accurately provided global and local classifications. A four-class OASIS-3 dataset was utilized for the experimental process and achieved a 99.68% accuracy. Abbas et al. [25] presented a CAD-ALZ method using the ConvMixer layer and blockwise fine-tuning technique to detect robust features. The author utilized an open-source dataset taken from Kaggle, including MR brain images, with an accuracy of 94%.

In addition, Rallabandi et al. [26] developed an Inception-ResNet wrapper model to classify AD using MRI and PET scans. This presented model utilized Fourier transformers and the DWT fusion method. For this experimental process, the OASIS dataset was utilized and achieved a 95.5% accuracy rate. The main drawback of this approach was the large number of parameters. Patil et al. [27] introduced an automated framework for detecting AD using MRI data. They trained the DenseNet-169 CNN model on 6000 brain MRI images of four classes. Using this technique, the framework attained an accuracy of 97%. Nguyen et al. [28] developed an ensemble learning approach by combining two deep learning models (3D ResNet and XGBoost). The experiments were conducted on MRI images taken from the ADNI dataset. By using this method, the authors were able to reach 96.2% of AUC. Hajamohideen et al. [29] introduced a novel neural network-based framework for the classification of AD. They presented the Siamese Convolutional Neural Network (SCNN) model using a triplet-loss function to transform the MRI images supplied as k-dimensional embeddings. During the process of transforming images into embedding space, the author applied both pre-trained and non-pre-trained techniques. The ADNI and OASIS datasets were used for testing, and accuracies were achieved at 91.83% and 93.85%, respectively.

In another study, Balaji et al. [30] implemented the hybrid deep learning approach to detect and classify AD using MRI, PET, and neuropsychological test scores. They used CNN and LSTM models and trained on the selected MRI datasets. Zhao et al. [31] introduced an Inheritable Deformable Attention Network (IDA-Net) to diagnose AD. They experimented using MRI images taken from the ADNI and AIBL datasets. By using this methodology, they achieved an accuracy of 92.7%. The disadvantage of this work is that deformed patches contain repetitive information, which weakens the model’s ability to extract global features. Odusami et al. [32] employed a multimodal fusion technique for Alzheimer’s disease detection. They combined data from the discrete wavelet transform (DWT) with deep features from a pre-trained VGG16 neural network. Their experiments, conducted using the ADNI dataset, achieved accuracy rates of 81.25% and 93.75%. However, the study faced a limitation due to redundant information during the fusion process, which led to increased time complexity.

Recent studies on Alzheimer’s disease (AD) prediction using MRI data have explored a variety of deep learning models, including transformers, ranging from conventional classifiers such as SVMs and Gaussian mixture models to advanced architectures such as CNNs, ResNets, Inception-ResNet, and hybrid deep learning networks. While these approaches have demonstrated promising performance, their focus has often been restricted to binary classification, distinguishing only between AD and non-AD states, which limits their utility in capturing disease progression across multiple stages. In addition, several works applied ensemble or hybrid designs, such as CNN–transformer combinations or multimodal fusions, but these were either computationally intensive due to feature-level concatenation or lacked the comprehensive interpretability of decision making. Models such as SCNNs with triplet loss and ConvMixer-based frameworks have shown improved classification accuracy, yet they often require significant computational resources and still struggle to generalize across diverse datasets.

To address these gaps, the present study introduces a novel network-level fusion architecture that integrates DenseNet201 and MobileNetV2 with a customized Vision Transformer for Alzheimer’s disease (AD) prediction. The CNN components capture local structural and textural features, while the transformer leverages global contextual dependencies, ensuring a more holistic feature representation. The architecture further employs Bayesian Optimization for hyperparameter initialization, mitigating the limitations of manual or random settings and enhancing reproducibility. A contrast enhancement preprocessing strategy is incorporated to emphasize critical neuroanatomical variations, improving model robustness across datasets. Finally, the proposed framework is validated on two benchmark datasets (ADNI and OASIS), and interpretability is ensured through Grad-CAM visualizations, bridging the gap between algorithmic prediction and clinical trust. This comprehensive design directly addresses the shortcomings of earlier studies by providing (i) multi-stage classification capability, (ii) efficient network-level fusion, (iii) automated hyperparameter tuning, and (iv) enhanced interpretability, ultimately offering a clinically relevant and computationally efficient solution for early AD detection.

## 3. Proposed AD Methodology

**Figure 2:**
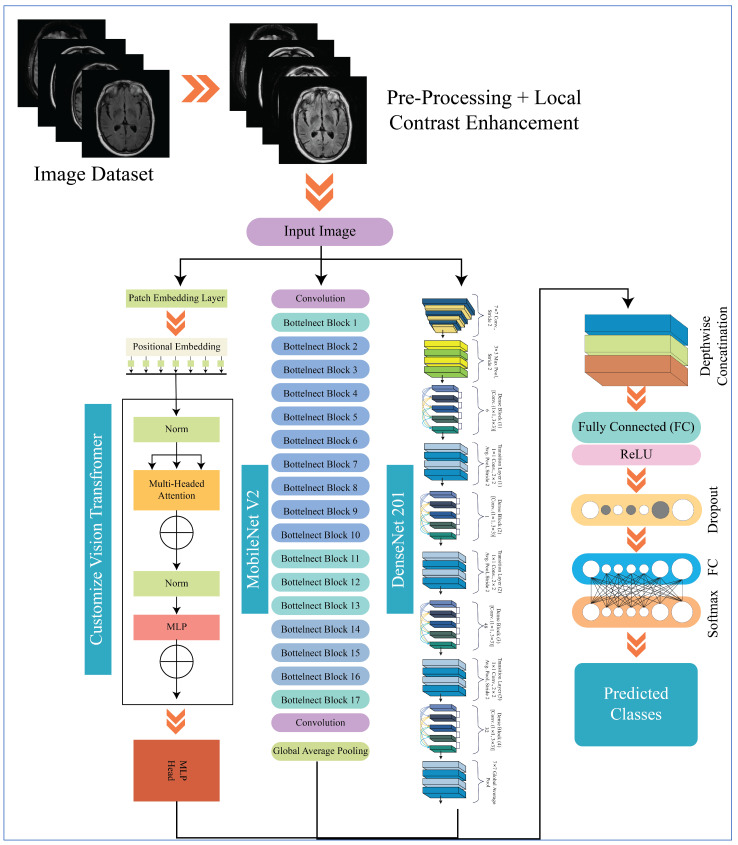
Proposed model for Alzheimer’s disease detection and classification.

Figure 2 illustrates the overall workflow of the proposed X-ViTCNN fused architecture for Alzheimer’s disease detection and classification. It combines contrast-enhanced MRI scans with a fused architecture of DenseNet201, MobileNetV2, and a customized Vision Transformer, enabling the complementary learning of local and global features for robust multi-stage AD prediction. The initial phase involves enhancing MRI scan contrast to accentuate subtle structural variations within brain tissue. The image contrast has increased at the early stage, and two pre-trained models are fused using a depth concatenation layer. The X-ViTCNN fused model is trained on contrast-enhanced images and further improved by fusing a custom ViT architecture using the same concatenation layer. The hyperparameters of the proposed X-ViTCNN fused architecture have been initialized using BO instead of random initialization. The final trained model features are passed to the Softmax classifier for classification. In addition, interpretable approaches, namely Saliency maps and grad-CAM, have been employed to enhance the explainability of the fused architecture. A detailed description of each step is discussed below.

### 3.1. Datasets

Two benchmark neuroimaging datasets (ADNI and OASIS) were used for the purpose of predicting Alzheimer’s disease through experimentation. The ADNI dataset includes a total of 21,430 MRI scans divided according to the four stages of the disease: cognitive normal (CN); early mild cognitive impairment (EMCI); late mild cognitive impairment (LMCI); and mild cognitive impairment (MCI). Comparatively, the OASIS dataset includes 18,668 images split into the four categories of the disease: non-demented (ND); very mild dementia (VMD); mild dementia (MLD); and moderate dementia (MRD). The description of participants involved in the ADNI dataset (Table 1) and their associated images (Table 2) can be found in the previously mentioned article. The models developed for this study utilized the complete dataset (i.e., 10,250 images) to train the models using the ADNI dataset. The original and utilized class distributions for both datasets and the train, test, and validate splits for each can be found in Table 3. An augmentation was completed on the original datasets due to class imbalances, especially concerning the moderate dementia category, so that the balanced training and valid evaluation of the proposed X-ViTCNN framework could take place.

**Figure 3:**
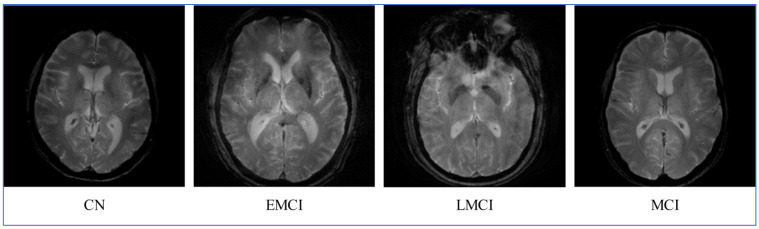
Sample images of the ADNI dataset for AD classification.

**Figure 4:**
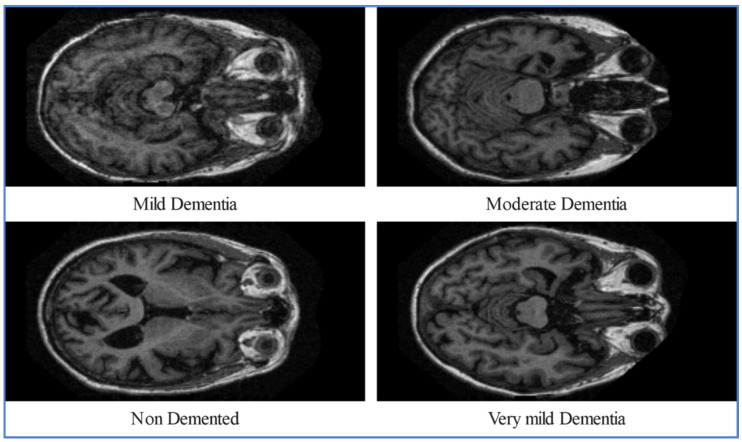
Sample images of the OASIS dataset for AD classification.

Figure 3 and Figure 4 depicts the class samples from representing MRI scans from the ADNI and OASIS datasets, respectively. All the images are two-dimensional MRI scans; each image includes a grayscale with dimensions of 224 × 224 × 3. These examples highlight the structural variations across disease stages that serve as an input for the proposed X-ViTCNN framework.

### 3.2. Preprocessing

Dataset preprocessing is a crucial step in computer vision, enhancing images and removing noisy factors to facilitate critical feature extraction and improve classification accuracy. In this study, we implemented a preprocessing step to produce high-quality images and boost the model’s ability to generalize across the varied brain images encountered in different patients. As a result, this reduces computational complexity and enables large-scale image analysis. In addition, these methods enhance quality control by detecting and eliminating artifacts and extraneous information from neuroimaging data, guaranteeing that only data of superior quality is used for analysis and diagnosis. In summary, image preprocessing methods are crucial in using neuroimaging data for Alzheimer’s disease (AD) research, diagnosis, and therapy monitoring.

**Figure 5:**
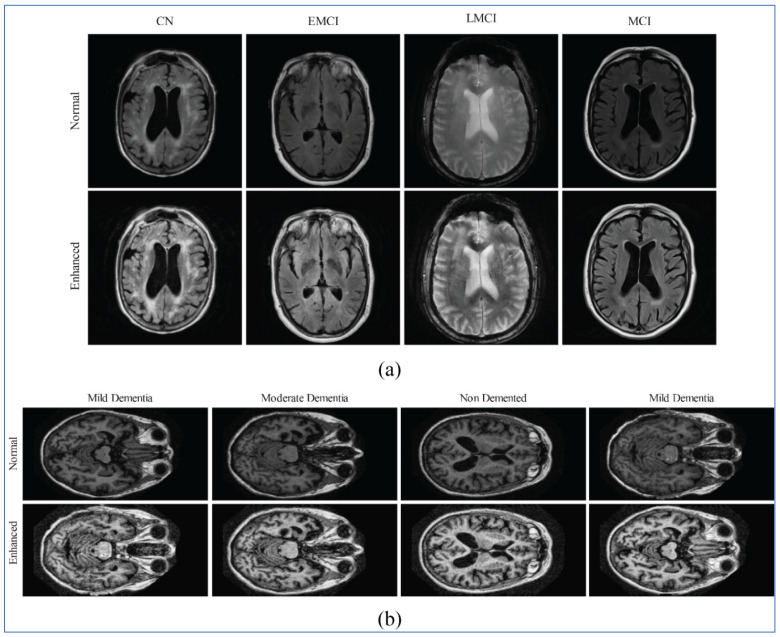
Sample MRI images from the ADNI and OASIS datasets with their corresponding contrast-enhanced versions. Subfigure (**a**) shows examples from the ADNI dataset, while (**b**) presents samples from the OASIS dataset. The contrast enhancement step improves the visibility of anatomical structures, facilitating better feature extraction for Alzheimer’s disease prediction.

In this work, we proposed a max-color mathematical formulation technique for contrast enhancement. In the initial step, the gray mean values of each color channel are computed by Equation (1).

(1) $${\tilde{K}}_{c}=\frac{1}{MN}\sum_{i=1}^{M}\sum_{j=1}^{N}K_{c}\left(i,j\right)$$

where M and N denote the row and column pixel values of the original image $K\left(i,j\right)$, and the max-gray scale mean is computed by Equation (2).

(2) $${\tilde{K}}_{ref}=max\left({\tilde{K}}_{r},\;{\tilde{K}}_{g},\;{\tilde{K}}_{b}\right)$$

where $max\left(\;\right)$ and ${\tilde{K}}_{ref}$ are the solution functions of max-value and max-ref mean, respectively. To further correct each channel in a piecewise fashion, the following Equation (3) has been employed as follows:

(3) $$\left\{\begin{array}{c} K_{cr}^{c}={\tilde{K}}_{ref}+\alpha \times \left(K_{c}-{\tilde{K}}_{c}\right),\;{\tilde{K}}_{ref}<K_{c}\\ K_{cr}^{c}={\tilde{K}}_{ref}-b\times \left({\tilde{K}}_{c}-K_{c}\right),\;{\tilde{K}}_{ref}\geq K_{c} \end{array}\right.$$

where α and b are the gain correction factors for each channel, and $K_{cr}^{c}$ is the color correction imager. A few resultant images are shown in Figure 5a. In this study, to address class imbalance, particularly for the moderate dementia class, several augmentation techniques were applied to enhance the MRI dataset. These techniques included horizontal and vertical flipping to introduce spatial variations, zooming by 20% to simulate different scales, and random rotations (±20 degrees) to ensure rotational invariance. Additionally, images were scaled between 80% and 120% of their original size to simulate variations in brain structure sizes, and elastic deformations were applied to mimic natural anatomical variations in brain tissue. These augmentation methods were carefully selected to preserve the real disease variability while preventing the introduction of synthetic patterns that may not reflect actual disease progression. Some example images are presented in Figure 5b.

### 3.3. Transfer Learning Models

Transfer learning models are a powerful approach in deep learning where a model trained on one task (usually with a large dataset) is reused and fine-tuned for another related task with less data. Pre-trained CNN architectures such as DenseNet201 and MobileNetV2 are employed as transfer learning models to extract discriminative local and structural features from MRI scans. These models serve as the backbone networks within the proposed X-ViTCNN framework, enabling efficient learning and improved generalization.

#### 3.3.1. DenseNet201

DenseNet201 provides interconnectivity among all neural network layers, allowing them to store maximum data flow and enabling the network to extract more features, thereby achieving high performance. This provides some benefits, including the resolution of the vanishing-gradient issue, enhanced feature propagation, the ability to utilize features, and a substantial decrease in the parameter count. Some levels, such as LMCI and EMCI, exhibit extremely similar characteristics, making differentiation difficult after several convolutional layers. The knowledge may degrade before reaching the desired destination due to the extended path between the input and output layers.

The objective of DenseNet201 was to tackle the accuracy problem caused by vanishing gradients in complex neural networks [33]. This makes an appropriate choice for AD categorization in this research. Furthermore, this model helps provide feature map concatenation to avoid feature loss and leads to better performance with reduced computational cost. DenseNet201 enables efficient training and superior performance by including concatenated feature maps from all previous levels in the next layer. The kth layer acquires feature maps from all preceding layers, generating its own feature maps using Equation (4).

(4) $$x_{k}=H_{k}\left(\left[x_{0},\;x_{1},\ldots x_{k}-1\right]\right)$$

The notation $\left[x_{0},x_{1},\ldots x_{k}-1\right]$ denotes the concatenation of feature maps generated in the corresponding layers. In addition to convolution, the composite function $H_{k}$ comprises batch normalization [34] and rectified linear unit (ReLU) [35]. Using Equation (5), the batch normalization layer can attain a uniform pattern of activations across the entire network.

(5) $$X_{i}=\frac{xi-\mu_{b}}{\sqrt{\sigma_{b}^{2}}}\;$$

The specified equations for the mini-batch mean ($\mu_{b}$) and variance ($\sigma_{b}^{2}$) are defined by Equations (6) and (7):

(6) $$\mu_{b}=\;\frac{1}{m}\sum_{i=1}^{m}x_{i}$$

(7) $$\sigma_{b}^{2}=\frac{1}{m}\sum_{i=1}^{m}{\left(x-\mu_{b}\right)}^{2}$$

Additionally, the mean and standard deviation were specified as trainable parameters in Equation (8).

(8) $$y_{i}=\gamma X_{i}+\beta \;$$

To improve the training process, the activation function of the ReLU was specified as $f\left(x\right)=max\left(0,x\right)$. For feature extraction, the convolution layer convolves the input representation with a kernel specified by Equation (9).

(9) $$C\left(i,j\right)=\sum_{u=-m}^{m}\sum_{v=-m}^{m}I\left(i-u,\;j-v\right)\times K\left(u,v\right)+b$$

where b denotes the term for bias. To introduce bottleneck layers, a convolution was performed before each 3 × 3 convolution. Therefore, the computational complexity in numerous layers is minimized. The selection of hyperparameters, including kernel size, stride, and padding, ensures that the dimensions of the feature map remain consistent throughout the block. A transition layer was implemented to reduce dimensions, consisting of 2 × 2 average pooling layers, batch normalization, and 1 × 1 convolution. As a result of the fine-tuning, the initial layers and network weights were transferred. Modifications were made to the final layers following the number of classes and additional convolutional layers. Adam optimizer [36] was utilized to optimize the learnable parameters to minimize the cross-entropy loss, as defined by Equation (10).

(10) $$ss=-\sum_{m=1}^{X}\sum_{n=1}^{Y}A_{mn}\ln\left(P_{mn}\right)$$

where *X* and *Y* indicate the number of samples and classes correspondingly. The notation $P_{mn}$ represents the predicted output while $A_{mn}$ represents the actual output. The parameters are modified by utilizing Equations (11)–(13).

(11) $$W_{i+1}=W_{i}-\frac{\alpha }{\sqrt{V_{i}}+\zeta }\;M_{i}\;$$

(12) $$M_{i}=\frac{m_{i}}{1+\beta_{1}^{i}}$$

(13) $$V_{i}=\frac{v_{i}}{1+\beta_{2}^{i}}$$

where bias-corrected mean is defined by $M_{i}$ and bias-corrected variance is defined by $V_{i}$.

#### 3.3.2. MobileNetV2

**Figure 6:**
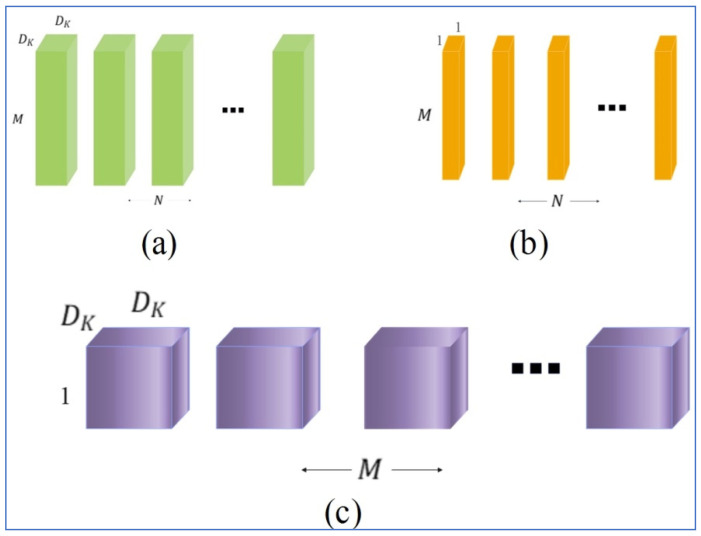
Visual description of convolutions. (**a**) Standard convolution filters; (**b**) pointwise convolution refers 1 × 1 convolutional filters in the context of depthwise separable convolution; (**c**) depthwise convolutional filters.

MobileNetV2 utilizes depthwise separable convolution layers, which are a kind of factorized convolutions that decompose standard convolution down into two stages: pointwise and depthwise. Each input channel receives one filter through the depthwise stage of MobileNetV2. The output produced in the depthwise stage is combined with one filter through a pointwise convolution (1 × 1), whereas standard convolutions take all filters from the depthwise stage and apply them in one step to create an output. By breaking a standard convolution into two parts (combining part and filtering part), depthwise separable convolutions greatly reduce both computation costs and model size. Figure 6 illustrates how a standard convolution (a) can be represented as depthwise convolution (c) and pointwise convolution 1 × 1 (b).

The input to standard convolutional layers can be expressed as feature map F with *D_F_* × *D_F_* × *M* dimensions where *D_F_* is both the height and the width of the feature map; M indicates the number of input channels (depth); *M* is the height and width of the output feature map; and N defines the number of output channels (depth), respectively.

The standard convolution layer can be described as a convolution kernel K with the size of *D_F_* × *D_F_* × *M* × *N*, where *D_F_* represents the size of the 2D convolution kernel and M and N are the number of input and output channels, respectively (as defined earlier). The output feature map for classical convolution is formed using a 1 × 1 stride (no offset) and can be mathematically characterized as per Equations (14) and (15).

(14) $$G_{k,l,n}=\sum_{i,j,m}K_{i,j,m,n}\times F_{k+i-1,l+j-1,m}$$

(15) $$D_{K}\times D_{K}\times M\times N\times D_{F}\times D_{F}\;$$

The cost of computation is determined by the number of input channels *M*, output channels *N*, kernel size $D_{K}\;$×$\;D_{K}$, and size of the feature map *D_F_* × *D_F_*. These variables and their interaction will be discussed in relation to the MobileNet architecture. For example, due to using depthwise separable convolutions, MobileNet is not reliant upon a relationship between the size of the kernel and the number of output channels generated. The purpose of the fundamental convolution operation is to combine features and to filter these combined features through the convolution kernel. The use of depthwise separable convolutions (also known as factorized convolutions or depthwise convolutions) allows for significant cost reductions since the filtering and combining processes of the convolution kernel can be separated into two distinct elements.

As an example of how to express a layer differently, let us take the separate convolutions that go into making up a separable convolution as an example. In this case of single-channel depthwise separable convolution, each channel has been assigned one filter. This creates a series of depthwise convolutions resulting in their combined output. In addition to this, a combination will then take place from the previous depthwise outputs to create pointwise convolutions (using a 1 × 1 convolution).

MobileNetV2 uses both batch normalization and ReLU nonlinearity for all two layers of each depthwise separable convolutions. A single 1 *dj* (operation) is applied to an individual input depth, and the example of a depthwise convolution is defined in Equation (16):

(16) $${\hat{G}}_{k,\;l,\;n}=\sum_{i,j,m}{\hat{K}}_{i,\;j,\;m,\;n}\times {\hat{F}}_{k+i-1,\;l+j-1,\;m}$$

where $\hat{K}$ is the $D_{K}\times D_{K}\times M$ depthwise convolutional kernel, and the $m_{th}$ channel of the filtered output feature map $\hat{G}$ is the result of applying $\hat{K}$ filter on F channel. The computational cost of depthwise convolution is defined in Equation (17):

(17) $$D_{K}\;\times \;D_{K}\;\times \;M\;\times \;D_{F}\;\times \;D_{F}\;$$

Although depthwise convolution is more efficient than standard convolution, it does not combine input channels to produce new features. To generate these new features, an additional layer is required. This layer uses a 1 × 1 (pointwise) convolution to perform a linear combination of the output from the depthwise convolution. This specific combination is referred to as depthwise separable convolution, as initially introduced in [37]. The computational cost of depthwise separable convolutions is expressed by Equation (18), which represents the sum of the depthwise and 1 × 1 pointwise convolutions:

(18) $$D_{K}\;\times \;D_{K}\;\times \;M\;\times \;D_{F}\;\times \;D_{F}\;+\;M\;\times \;N\;\times \;D_{F}\;\times \;D_{F}$$

By conceptualizing convolution as a two-step procedure involving filtering and combining, the formulation is defined by Equation (19).

(19) $$\frac{D_{K}\times D_{K}\times M\times D_{F}\times D_{F}+M\times N\times D_{F}\times D_{F}}{D_{K}\times D_{K}\times M\times N\times D_{F}\times D_{F}}\;\;=\frac{1}{N}+\frac{1}{D_{K}^{2}}$$

According to normal convolutions, MobileNetV2 utilizes 8 to 9 times less computing using 3 × 3 depthwise separable convolutions. Although depthwise convolutions require minimal computational resources, incorporating spatial dimension factorization, as demonstrated in references [38,39], does not yield substantial computational savings.

### 3.4. Custom ViT Architecture

Let *S* be an arbitrary collection of *r* remote sensing images as determined by {*X_i_*,*Y_i_*}:{*i* = 1 to *r*}. Each *X_i_* is a separate, individual image, while *Y_i_* corresponds to the class label assigned to *X_i_*. The class assignment can take any value from *Y_i_* ={1, 2, …, *m*}, where *m* = the number of classes specified for this collection of images.

The ViT model is focused on locating image patches and the corresponding semantic class label associated with each image patch (ViT model). The ViT is designed using only the vanilla transformer architecture (Transformer) as previously described [40]. The recent popularity of the transformer architecture has stemmed from the ViT model’s remarkable success in task areas such as NLP, etc. [41]. One of the characteristics of the transformer architecture is that it comprises an encoder and decoder with a sequence of sequential data being able to be processed simultaneously, rather than through a recurrent neural network-type approach. The transformer architecture’s self-attention mechanism has aided the successful use of transformer-based approaches for modeling with long-term relationships between the sequence elements.

The ViT has been proposed to complement the traditional transformer for image categorization. Its main goal is to apply these techniques across various modalities beyond text, without relying on architecture specific to the data. The transformer encoder module in the ViT handles classification, transforming a sequence of image patches into a semantic label. Furthermore, unlike standard CNN architectures that typically use filters with a local receptive field, the attention mechanism allows it to focus on different parts of the image and combine information from the entire image.

The Vision Transformer (ViT) has been introduced as an extension to the classic transformer for the classification of images. The main purpose of ViT is to deliver transformer-based solutions to every input type besides textual inputs, without requiring the architecture to conform to the data characteristics. The transformer encoder component in the ViT performs the classification of input image patch sequences into a semantic label. In comparison to traditional convolutional neural networks (CNNs) that typically implement local filter-sized receptive fields, the ViT utilizes attention mechanisms that enable it to attend to various locations in the image and amalgamate all information from the complete input image.

**Figure 7:**
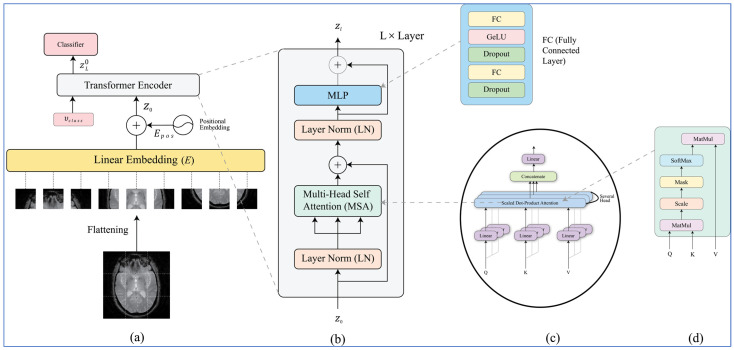
Custom Vision Transformer (ViT) architecture used in the proposed X-ViTCNN framework. (**a**) Input MRI image is divided into fixed-size patches and embedded into a sequence of tokens. (**b**) Positional encoding is added to preserve spatial information. (**c**) The token sequence is processed through stacked transformer encoder layers with multi-head self-attention and feed-forward networks. (**d**) The final classification head outputs the predicted Alzheimer’s disease stage.

The overall structure of the model is displayed in Figure 7. The model consists of an embedding layer, encoder module and classification head. Initially, a non-overlapping patching strategy is applied on to the training image X, where each extracted patch represents a separate token to be the input to the transformer architecture. Each patch is determined to have the dimensions of c × p × p, being extracted from an image of dimensions c × h × w, where h represents height, w represents width, and c represents the number of channels. The patches are segregated into the sequence x1, x2, …, xn, with n defined as hw/p^2^. Typically, the size p of the patch is 16 × 16 or 32 × 32.

We chose DenseNet201 and MobileNetV2 in combination with the ViT for their complementary strengths in feature extraction. DenseNet201 excels in capturing local structural features due to its dense connectivity pattern, while MobileNetV2 provides a lightweight architecture that is computationally efficient, making it suitable for resource-constrained environments. The customized ViT, on the other hand, is designed to capture long-range dependencies and global features, making it an ideal candidate to supplement the CNN-based models. This combination allows the model to capture both fine-grained local features and global contextual information, which is critical for accurately predicting multi-stage Alzheimer’s disease from MRI scans.

The ViT, a core component of the X-ViTCNN architecture, was tailored for AD prediction using MRI scans. It features 12 encoder layers and an embedding dimension of 768, which balances model complexity and computational efficiency. This configuration allows the model to capture both fine-grained local and long-range global features essential for accurate AD classification. A 16 × 16 patch size was chosen to balance computational cost and the preservation of spatial details in the MRI scans. Smaller patch sizes would increase computational demands, while larger ones could miss important features. The selected patch size effectively captures both local and global information. A key design choice was the use of a custom ViT architecture rather than a pre-trained ViT backbone. This decision was made because the MRI scans in Alzheimer’s research have unique characteristics that may not align well with general pre-trained models designed for natural images. A custom ViT allows for better adaptation to these specific features and provides more control over the model’s learning process. Additionally, pre-trained models typically require large and diverse datasets like ImageNet, which are not always representative of the smaller, specialized datasets in medical imaging. The custom ViT architecture was specifically tailored for the challenge of Alzheimer’s disease prediction, ensuring more accurate and relevant feature extraction from MRI data.

#### 3.4.1. Model Parameters and Specification

Table 4 summarizes the parameters, core components, strengths, and limitations of the models used in this study, including DenseNet201, MobileNetV2, the customized Vision Transformer, and the proposed fusion architecture.

#### 3.4.2. Linear Embedding Layer

A trained embedding matrix E converts the digitized patches into a d-dimensional vector before they enter an encoding stage of transformation. For the classification task, these embedded representations are also combined with an additional trainable classification token *υ_class_*. The transformer analyzes the entire collection of patches at once and does not address the order in which they are arranged or sequenced.

To allow for the preservation of the original image’s spatially arranged patch locations within the input, positional information has been encoded into the patch representations. An example of the encoded patch sequence that includes the initial classification token (referred to as *z*_0_) is shown in Equation (20).

(20) $$z_{0}=\left[\upsilon_{class};\;x_{1}E;\;x_{2}E;\ldots ;\;x_{n}E\right]+E_{pos},\;E\;\epsilon \;R^{\left(p^{2}-c\right)\times d},\;E\;\epsilon \;R^{\left(n+1\right)\times d}\;$$

#### 3.4.3. Vision Transformer Encoder

The *z*_0_ sequence will be fed into the transformer encoder for processing. As illustrated in Figure 6b, we have constructed the transformer encoder using n identical layers. Each layer contains two primary subcomponents; first, one fully connected feedforward MLP block consisting of two fully connected ML structures separated with a GELU activation function, and then one multi-head self-attention (MSA) block as defined in Equation (21) and MLP block is defined in Equation (22). Finally, we apply layer normalization (LN) and the incorporation of a residual skip connection between both of the two subcomponents of the encoder on a per-layer basis.

(21) $$z_{l}^{\prime }=MSA\left(LN\left(z_{l-1}\right)\right)+z_{l-1},\;l=1\ldots L$$

(22) $$z_{l}=MLP\left(LN(z_{l}^{\prime }\right))+z_{l}^{\prime },\;l=1\ldots L$$

The last phase of the encoder includes transferring the first element of the sequence $z_{L}^{0}$ to an external head classifier to predict the class label.

(23) $$y=LN\left(z_{L}^{0}\right)$$

The encoder has a structural component called an MSA block that is the core of the transformer architecture. The purpose of the MSA block is to evaluate how much one patch embedding is connected to another within a patch embedding sequence. The MSA block consists of several components: a self-attention layer, a linear layer, a concatenating layer (which combines multiple attention outputs), and a final linear layer as illustrated in Figure 6c. The overall attention weight for the input sequence (i.e., *z*) is calculated as a weighted average of every value present in the series. The SA head computes attention weights via a dot product between the Q, K and V for each position of the input sequence as described in Figure 6. The results yield Q, K and V for each position of the input sequence via the matrix elements of UKQV where each UKQV is generated via an element-by-element multiplication of the input sequence and the learnt matrices UKQV (cf. Equation (23)). The relative value of an element to all other elements in the input sequence is computed using the dot product between the Q vector of the element and the K vector of each other element in the input series. The resulting values indicate the intrinsic relative value of different portions in the series. In Equation (24), the scaled dot product values are passed through a softmax function. To identify which patch has the greatest attention score, each patch embedding vector value is multiplied by the output of the softmax function, again as described in Equation (25). In addition to this, the SA block’s scaling of dot products uses the key dimension for determining how each of them gets scaled for the purpose of creating the SGEMM operation as defined in Equation (26). These equations explain the full set of work performed to locate points with high attention by using attention scores:

(24) $$\left[Q,\;K,V\right]=zU_{QKV},\;U_{QKV}\;\epsilon \;R^{d\;\times 3D_{K}}$$

(25) $$A=softmax\left(\frac{QK^{T}}{\sqrt{D_{K}}}\right),A\epsilon \;R^{n\times n}\;$$

(26) $$SA\left(z\right)=A\times V\;$$

To independently calculate the scaled dot product attention, the MSA block will apply the same operation for each of the *h* heads. Each of the attention head’s individual outputs will be merged together and subsequently projected into the desired dimension via a feed forward layer, utilizing learnable weights W. This operation is reflected in Equation (27):

(27) $$MSA\left(z\right)=Concat\left(SA_{1}\left(z\right);SA_{2}\left(z\right);\ldots SA_{h}\left(z\right)\right)W,\;W\;\epsilon \;R^{h.D_{K}\;\times D}\;$$

Table 5 presents a comparative overview of the adopted architectures, focusing on their structural design, feature extraction capabilities, and relative computational complexity.

#### 3.4.4. Network-Level Fusion

The designed custom ViT and pre-trained networks are fused into a single network to improve AD prediction’s learning capability. First, the DenseNet201 and MobileNetV2 architectures are fused using a depth concatenation (DC) layer. After that, the X-ViTCNN fused architecture is further concatenated with the custom ViT model. The DC layer is again used to fuse both these networks. After the fusion process, additional layers were added, such as dropout, softmax, and classification output. Mathematically, this process is defined as follows:

Given the global average pool layer of DenseNet201 defined by $L_{gap}$ and average pool layer MobileNetV2 defined by $L_{avg}$, the depth concatenation is defined in Equation (28).

(28) $$Depth\left(1\right)=DC\left(L_{gap},L_{avg}\right)$$

The output of Depth (1) is further fused with a custom ViT model layer named MLP to refine the proposed model results. The working of the final fusion as follows:

(29) $$Depth\left(2\right)=DC\left(Depth\left(1\right),L_{ViT}\right)$$

After $Depth\left(2\right),$ we added a few layers such as fully connected, dropout, fully connected, softmax, and classification output layer as described in Equation (29).

#### 3.4.5. Model Training for AD Classification

The proposed X-ViTCNN fused model was trained on selected datasets. Several hyperparameters, such as initial learning rate, momentum, regularization factor, epochs, and optimizer, are required in the training process. Researchers typically set hyperparameters based on the existing literature knowledge; however, this approach is not ideal. We employed a Bayesian Optimization (BO) approach to initialize hyperparameters in this work. With BO, the initial learning rate was determined to be 0.000121, the momentum was set at 0.705, the batch size was 128, and the optimizer used was SGD. The trained model is later utilized for the activation function which extracts features and passes to the softmax classifier for the final classification results.

In this study, Bayesian Optimization (BO) was used to fine-tune the hyperparameters of the X-ViTCNN architecture. The optimized hyperparameters included learning rate, momentum, and batch size. The search space for learning rate was between 1 × 10^−6^ and 1 × 10^−2^, momentum between 0.1 and 0.9, and batch size between 16 and 256. The BO process was carried out over 50 iterations, using the Expected Improvement (EI) acquisition function to balance exploration and exploitation. The optimization was stopped after five consecutive iterations with less than 0.5% improvement in validation accuracy.

#### 3.4.6. Interpret Fused Model

This work utilized the Grad-CAM visualization tool for the explainable X-ViTCNN fused model. For this purpose, we utilized the Grad-CAM technique. Grad-CAM: One further variant of CAM that has been expanded upon is the Gradient Class Activation Mapping (Grad-CAM) [42]. The approach is tailored to a particular CNN architecture in CAM, where the GAP layer feeds straight into a softmax layer. Conversely, Grad-CAM allows you to inspect any convolutional layer by utilizing backpropagation to calculate the gradient and applying GAP to weigh each feature map output in that layer as:

(30) $$f\upsilon_{f}=\frac{1}{P}\sum_{i,j}\frac{\partial S_{c}}{\partial FV_{f}}$$

(31) $$gCAM=ReLU\left(\sum_{f}f\upsilon_{f}\times FV_{f}\right)$$

In Equations (30) and (31), P denotes total pixels, $S_{c}$ is the class score, and $FV_{f}$ deotes the feature map activation of feature f. Before passing the feature map outputs via a ReLU function, which ensures that only positive contributions to the class are presented, the output is weighted and added. Based on these maps, the image’s strong points are captured.

## 4. Results and Discussion

In this section, the results of the proposed X-ViTCNN model are showcased and examined. The experimental setup is described in detail, followed by an exploration of the evaluation metrics used. Additionally, the model’s performance is assessed using the ADNI and OASIS datasets, and its interpretability is analyzed through Grad-CAM. The next subsection emphasizes the clinical significance of the results. Lastly, an ablation study is performed to confirm the effectiveness of the proposed fusion architecture.

### 4.1. Experimental Setup

This section outlines the experimental configuration. Several hyperparameters are initialized for each network and the final X-ViTCNN fused network. The BO is employed to initialize these hyperparameters, such as an initial learning rate of 0.000121, momentum 0.705, batch size 128, Adam optimization approach, and softmax feature activation function. The loss is calculated using the categorical cross-entropy loss function. All experiments are conducted on a system running Windows 11, Python 3.11.5, CUDA 12.2, TensorFlow 2.14.0, Matplotlib 3.8, SciPy 1.11.3, NumPy 1.20.3, an Intel Core i7 CPU with 32 GB RAM, and an NVIDIA RTX TITAN with NVIDIA GeForce Graphics Driver 537.58. Each experiment is repeated 50 times, and the results reported are the average accuracies.

### 4.2. Performance Metrics

A confusion matrix is a fundamental tool for assessing the performance of classification systems. This matrix is square-shaped, featuring four rows and four columns that correspond to the various categories within the dataset. It displays the count of correctly and incorrectly identified samples from the test group. True-positive (TP) samples are positioned along the main diagonal, while true-negative (TN) and false-negative (FN) samples are found in the other cells. The system’s functionality is evaluated using specific equations, with variables derived from the confusion matrix. Another metric for assessing a model’s accuracy is the proportion of correctly predicted pixels to the total number of pixels. This can be represented by the Equations (32)–(36) based on the confusion matrix:

(32) $$Accuracy=\;\frac{TP+TN}{TP+TN+FN+FP}$$

(33) $$Recall=\frac{TP}{TP+FN}$$

(34) $$Specificity=\frac{TN}{TN+FP}$$

(35) $$Precision=\frac{TP}{TP+FP}$$

(36) $$F1-Score=\frac{2\times Recall\times Precision}{Recall+Precision}$$

### 4.3. Model Evaluation and Assessment

We performed the experimental process for each network and proposed an X-ViTCNN fused architecture. The above-listed parameters are computed to validate each network. A total of 70% of the dataset images are utilized for the training, whereas the remaining 30% are used for the testing.

- Using ADNI Dataset

**Figure 8:**
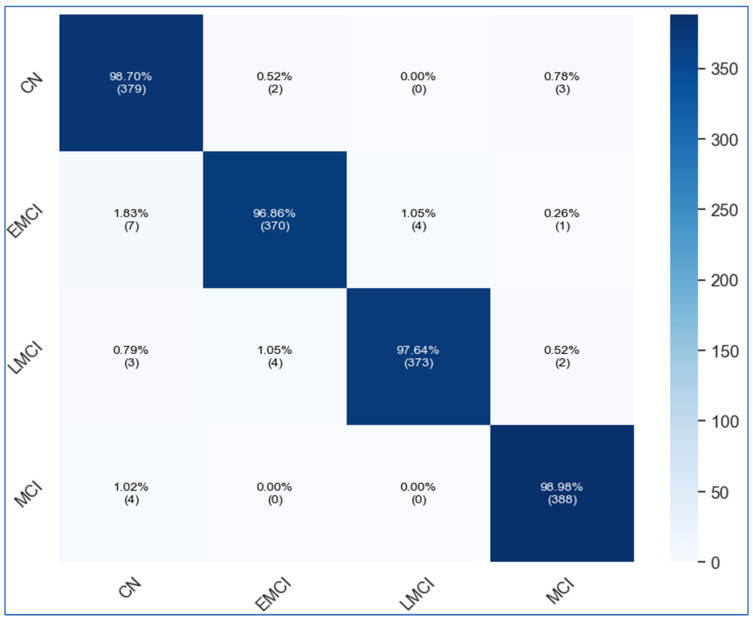
Confusion matrix of proposed X-ViTCNN fused architecture on ADNI dataset.

**Figure 9:**
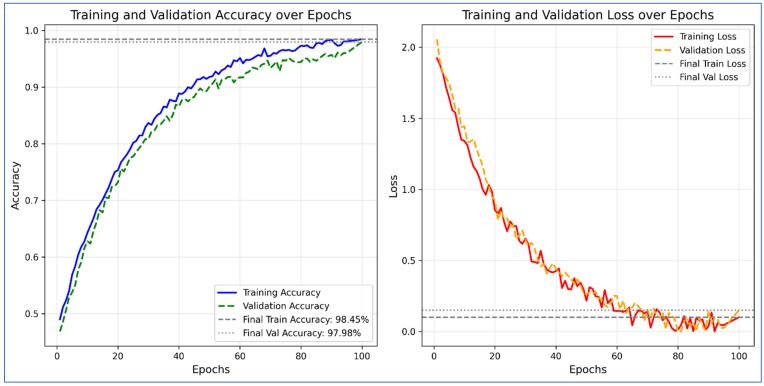
Training and validation accuracy and loss of proposed X-ViTCNN architecture on the ADNI dataset.

Table 6 displays the results of the ADNI dataset using the proposed architecture. In this table, the results are also computed for each network, including fine-tuned DenseNet201, fine-tuned MobileNetV2, and custom ViT. For fine-tuned DenseNet201, the obtained accuracy is 64.25, whereas the precision and specificity values are 78.65 and 88.08%, respectively. For the fine-tuned MobileNetV2 architecture, the obtained accuracy value of 56.5%, whereas the precision and specificity values were 63.87% and 85.49%, respectively. The proposed custom ViT architecture achieves an improved accuracy of 91.33%, with precision and specificity values of 94.77% and 97.67%, respectively. The proposed X-ViTCNN fused architecture achieved an accuracy of 97.98%, surpassing that of individual networks. Moreover, the precision rate of the proposed X-ViTCNN fused architecture is 99.33, the recall rate is 97.42, and the specificity value is 99.67%. Figure 8 shows the confusion matrix of the proposed X-ViTCNN fused architecture. This figure shows that the correct prediction rates for the CN class, EMCI class, LMCI class, and MCI class are 98.70%, 96.86%, 97.4%, and 96.98%, respectively. Figure 9 shows the training and validation accuracy of the proposed X-ViTCNN fused architecture on the ADNI dataset.

B. Using OASIS Dataset

**Figure 10:**
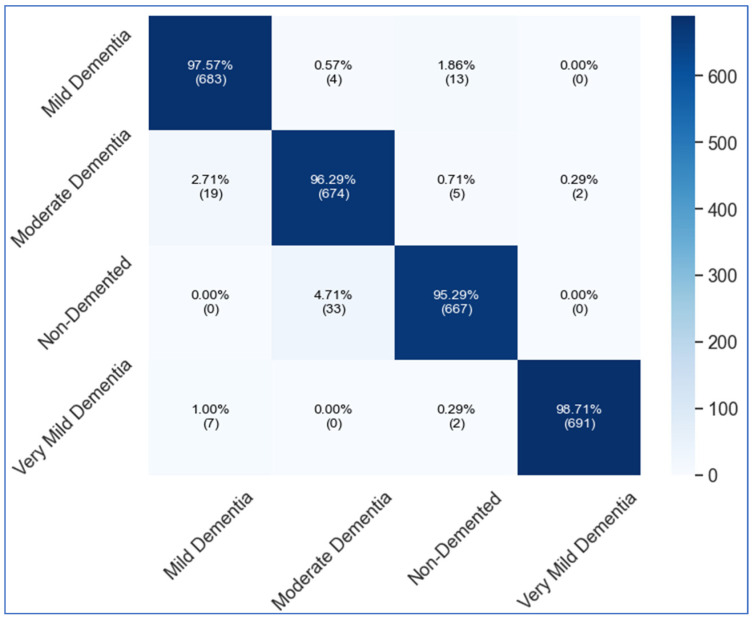
Confusion matrix of the proposed fused CNN on the OASIS dataset.

**Figure 11:**
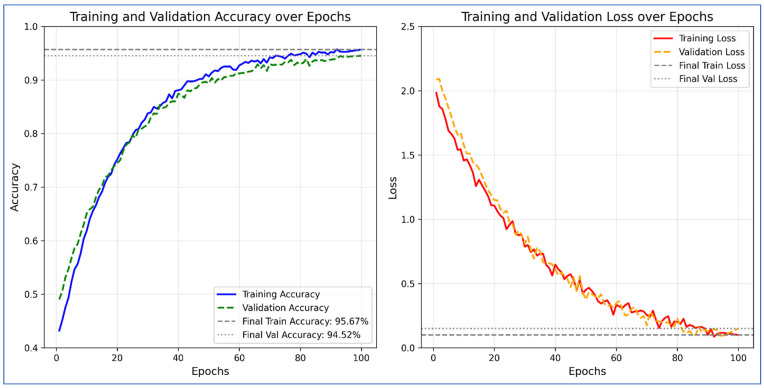
Training and validation accuracy for the proposed methodology on the OASIS dataset.

Table 7 elaborates on the results of the OASIS dataset. Table 2 describes the experimental study utilizing fine-tuned DenseNet201, fine-tuned MobileNetV2, proposed custom ViT, and fused CNN architecture. DenseNet201 initially achieved 67.45% accuracy, while other characteristics such as precision, recall, specificity, and F1-score achieved 81.22%, 67.98%, 91.33%, and 66.43%, respectively. MobileNetV2 achieved an accuracy of 54.02%, while the precision rate, recall rate, specificity, and F1-score are 62.46%, 55.67%, 80.46%, and 53.16%, respectively. The proposed custom ViT model obtained an improved accuracy of 90.88%, with precision, recall, specificity, and F1-score values of 94.06%, 89.98%, 96.33%, and 90.12%, respectively. Lastly, the proposed X-ViTCNN fused architecture is employed, yielding an improved accuracy of 94.52%. The other measures of this architecture are precision rate, recall rate, specificity, and F1-score of 97.99%, 96.23%, 98.16%, and 94.17%, respectively. Figure 10 shows the confusion matrix of the proposed X-ViTCNN fused architecture. This figure shows that the correct prediction rate of the mild dementia class is 97.57%, the moderate dementia class is 96.29%, the non-demented class is 95.29%, and the very mild class is 96.71%, respectively. Figure 11 shows the training and validation curve of the proposed X-ViTCNN fused architecture on the OASIS dataset.

Table 8 presents the performance comparison of the proposed X-ViTCNN model with other architectures including DenseNet201, MobileNetV2, and Custom ViT, on a naturally balanced cohort from the OASIS dataset. The X-ViTCNN model outperforms all other models across key evaluation metrics, including accuracy, precision, recall, specificity, and F1-score, with an accuracy of 94.52% and an F1-score of 94.17%. In contrast, DenseNet201 and MobileNetV2 show significantly lower performance, particularly in accuracy and precision, indicating their limitations when used independently for multi-stage Alzheimer’s disease classification. While custom ViT performs better than the CNN-only models, it still falls short of the X-ViTCNN model due to the absence of local feature extraction from CNNs. This comparison highlights the superior performance and generalizability of the X-ViTCNN architecture.

### 4.4. Model Interpretability Using Grad-CAM XAI

**Figure 12:**
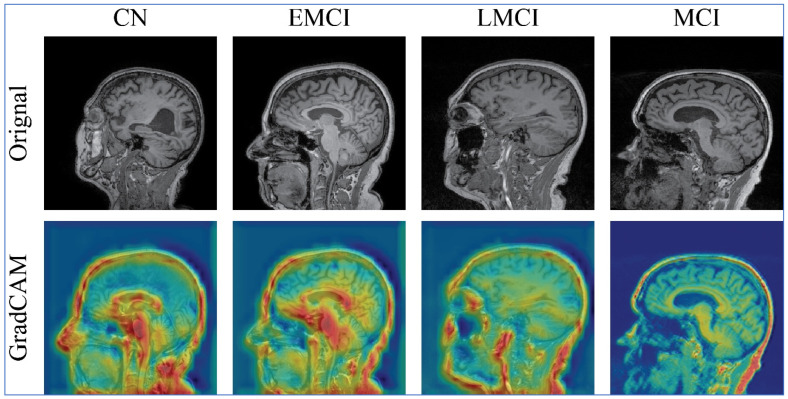
Grad-CAM-based interpretability of the proposed X-ViTCNN fused architecture on the ADNI dataset.

The visual effectiveness of the proposed model is assessed through the Grad-CAM XAI method. Figure 12 illustrates the qualitative analysis of this technique using the proposed model. The model produces activation maps that are depicted in various colors. It is clear that the attention maps for each category successfully pinpoint the object instances belonging to the same category, irrespective of the brain image’s shape and size. The Grad-CAM technique demonstrates that the model can accurately identify the disease region.

### 4.5. Ablation Study

On the ADNI dataset, the ablation results presented in Table 9 show that individual CNNs (DenseNet201 and MobileNetV2) perform weakly, while the custom ViT achieves higher accuracy but still falls short of the fused model. CNN fusion without the ViT improves performance yet misses global dependencies. Removing contrast enhancement reduces accuracy, confirming the benefit of preprocessing in highlighting structural brain details. Similarly, excluding Bayesian Optimization lowers performance due to suboptimal hyperparameters, comparing network-level fusion against other fusion strategies like element-wise addition and attention-based weighting. It demonstrates that depth concatenation outperforms alternative fusion methods by preserving detailed local and global features, achieving the highest accuracy of 97.98% on the ADNI dataset. Alternative fusion strategies and larger ViT patches also degrade results by losing discriminative features. Overall, the X-ViTCNN fused model achieves the highest accuracy, demonstrating the combined effect of all its components.

On the OASIS dataset, Table 10 shows a similar trend: the DenseNet201 and MobileNetV2 baselines remain weak, whereas the custom ViT performs better but still lags behind the fused model. CNN fusion of the two CNNs alone improves results, but cannot match ViT’s global feature learning. Removing contrast enhancement decreases accuracy, showing its role in improving MRI clarity, while excluding Bayesian Optimization again reduces generalization. Summation fusion and larger patch sizes slightly degrade performance, emphasizing the importance of depth-concatenation and fine-grained patches. The full X-ViTCNN fused architecture achieves the best results, confirming that each component makes a critical contribution to Alzheimer’s classification.

### 4.6. Discussion and Clinical Implications

**Figure 13:**
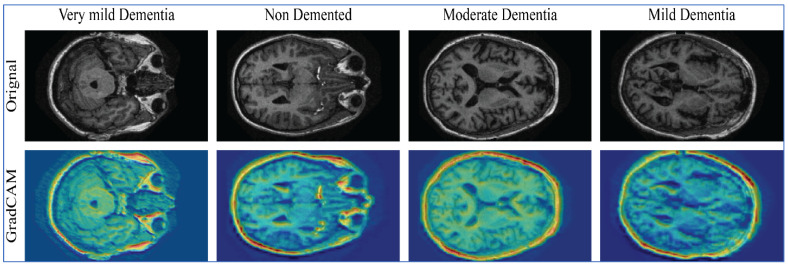
Grad-CAM-based interpretability of the proposed X-ViTCNN fused architecture on the OASIS dataset.

This section provides a detailed discussion of the proposed work. Figure 2 displays the proposed X-ViTCNN fused architecture for AD classification and prediction. It shows that the contrast of the original images has been enhanced using a max-color technique, as shown in the visual results of Figure 5. The results are calculated for each considered model across the evaluation metrics as presented in Table 6 and Table 7. The confusion matrices for proposed X-ViTCNN fused architecture are shown in Figure 8 and Figure 10. Additionally, training and validation curves for accuracy and loss are plotted in Figure 9 and Figure 11. Furthermore, the XAI-based Grad-CAM heatmaps are presented in Figure 12 and Figure 13. Across both datasets, X-ViTCNN consistently outperforms all baselines and prior fused variants. On ADNI, the accuracy reaches 97.98% (precision 99.33%, recall 97.42%, specificity 99.67%); on OASIS, it is 94.52% (precision 97.99%, recall 96.23%, specificity 98.16%).

These gains are explained by the network-level fusion of DenseNet201 and MobileNetV2 with a customized ViT: CNN branches contribute fine-grained, local morphometric cues (e.g., hippocampal and cortical patterns), while the ViT captures global dependencies across distant anatomical regions critical for subtle stage differentiation. The ablation studies corroborate this mechanism: (i) either CNN alone underperforms; (ii) CNN-only fusion improves but still trails the full model; and (iii) removing contrast enhancement or Bayesian Optimization yields measurable drops, showing that enhancing local signal-to-noise ratio and principled hyperparameter search both translate into better generalization. Class-wise confusion matrices indicate robust recognition across stages (e.g., high true-positive rates for CN, EMCI/LMCI, and MCI in ADNI, and balanced detection of non-demented individuals through moderate dementia in OASIS), suggesting that the model avoids collapsing borderline categories. Training/validation curves further show stable optimization without overfitting, aligning with the use of dropout, depth-concat regularization, and BO-selected learning rates and batch sizes. Methodologically, X-ViTCNN also addresses common pitfalls in the literature: (a) it avoids feature-level concatenation that often introduces redundancy and heavy tensors by fusing at the network level, (b) it pairs transparent interpretability (Grad-CAM) with high accuracy, and (c) it demonstrates cross-dataset robustness despite differences in acquisition and class balance (notably the augmented moderate dementia cohort in OASIS).

In addition to stage-wise classification, we further examined whether the neuroanatomical information captured by X-ViTCNN reflects clinical symptom severity, as stated in the Introduction. An MRI-derived severity score was computed from the model’s softmax outputs using an ordinal weighting of disease stages (CN = 0, EMCI = 1, LMCI = 2, MCI = 3). Spearman’s rank correlation analysis revealed a strong and statistically significant association between the MRI-derived severity score and clinical disease stage on both datasets (ADNI: ρ = 0.82, *p* < 0.001; OASIS: ρ = 0.79, *p* < 0.001). This finding quantitatively confirms that the structural brain representations learned by the model align closely with symptom progression, thereby supporting its use for severity-aware prediction of Alzheimer’s disease.

The results indicate that X-ViTCNN can support early, multi-stage triage by reliably separating CN from EMCI/LMCI/MCI and stratifying dementia severity, which is vital for timely referral, care planning, and recruitment into disease-modifying trials. Its high specificity reduces unnecessary follow-ups, and its high recall helps limit missed cases—both essential for population-level screening where prevalence is rising. The Grad-CAM maps localize disease-relevant regions, providing visual evidence that can increase clinician trust, facilitate radiology–neurology dialog, and serve as educational feedback for trainees. In longitudinal care, consistent stage-wise predictions can assist in monitoring progression and evaluating therapy response. In settings with limited expert availability, the relatively lightweight MobileNetV2 branch within the fusion suggests deployable variants (e.g., pruning/quantization of the full model) for resource-constrained clinics. From a workflow perspective, X-ViTCNN can be integrated as a decision-support layer in PACS/RIS: (i) pre-read prioritization (flagging suspected EMCI/LMCI for expedited review), (ii) structured reporting (auto-generated probability scores and attention overlays), and (iii) quality control (alerts when image quality/contrast fall below learned thresholds). Before routine adoption, institutions should establish site-specific validation, bias and calibration audits (by age/sex/scanner), as well as governance for human-in-the-loop sign-off. With these safeguards, the model’s combination of accuracy, robustness, and interpretability positions it as a clinically actionable aid for MRI-based Alzheimer’s assessment.

### 4.7. Comparison Analysis

Table 11 compares the performance of the proposed X-ViTCNN framework with several fine-tuned pre-trained neural networks, including DenseNet201, MobileNetV2, ResNet101, ResNet50, Inception-ResNet, and the customized Vision Transformer. While individual models achieved moderate accuracy ranging from 49.80% to 67.45% on OASIS and 51.29% to 64.25% on ADNI, the proposed X-ViTCNN outperformed all baselines, attaining 97.98% accuracy on ADNI and 94.52% on OASIS. These findings highlight the effectiveness of network-level fusion in harnessing the complementary strengths of CNNs and the Vision Transformer for reliable multi-stage prediction of Alzheimer’s disease.

The proposed fused X-ViTCNN architecture is compared with recent techniques on selected datasets as presented in Table 12. It can be observed that Hazarika et al. [23] used a hybrid CNN model and performed experiments on the ADNI dataset, achieving an accuracy of 93.67%. Zhao et al. [31] introduced the IDA-NET model, attaining an accuracy of 85.20%. Li et al. [43] introduced the EfficientNet B2 and GCM-EB2 models, achieving an accuracy of 92.42%. Qu et al. [44] introduced a UNB-GCN architecture and achieved an improved accuracy of 93.90%. The proposed fused architecture achieved 97.98% and 94.52% accuracy on the ADNI and OASIS datasets, respectively.

### 4.8. Statistical Analysis

In Table 13, the X-ViTCNN model achieved impressive performance on the ADNI dataset, with an average accuracy of 97.98% across 50 repeated experiments. The standard deviation for accuracy was 0.85%, and the 95% confidence interval ranged from 97.10% to 98.86%, highlighting the model’s consistent performance. Precision was similarly high at 99.33% with a standard deviation of 0.77%, and the confidence interval for precision ranged from 98.47% to 100.19%. The recall achieved was 97.42%, with a standard deviation of 0.91%, and the confidence interval ranged from 96.50% to 98.34%. Specificity was particularly strong at 99.67%, with a narrow confidence interval of 98.48% to 100.02%. The F1-score was 97.12%, with a standard deviation of 0.82%, and the confidence interval for the F1-score ranged from 96.29% to 97.95%. Paired t-tests showed statistically significant improvements over the baseline models, with a *p*-value of <0.001, confirming that the model’s high performance is not due to random variation but rather reflects its effectiveness in AD classification.

On the OASIS dataset, the X-ViTCNN model also demonstrated robust performance, achieving an accuracy of 94.52% with a standard deviation of 1.23%. The 95% confidence interval for accuracy ranged from 93.26% to 95.78%, indicating strong stability in its predictions. Precision was 97.99%, with a standard deviation of 1.15%, and the confidence interval ranged from 96.02% to 99.96%. The recall on OASIS was 96.23%, with a standard deviation of 1.09%, and the confidence interval for recall ranged from 95.12% to 97.34%. Specificity was 98.16%, with a confidence interval of 97.23% to 99.09%, and the F1-score was 94.17%, with a standard deviation of 1.12%, and a confidence interval of 93.02% to 95.32%. Statistical analysis using paired t-tests showed a *p*-value of <0.01, indicating that the performance improvements over the baseline models are statistically significant and not within the noise margins, further validating the model’s robustness and generalizability across different datasets.

## 5. Conclusions, Limitations and Future Work

This research introduced X-ViTCNN, an innovative fusion framework at the network level that combines DenseNet201, MobileNetV2, and a tailored Vision Transformer, to predict the various stages of Alzheimer’s disease using MRI images. Initially, the model enhances and flips images to increase dataset diversity. We fused two pre-trained models using a depth concatenation layer; however, their performance was insufficient. Therefore, we designed a custom ViT architecture that combines information from fused pre-trained models through a depth concatenation layer. The X-ViTCNN fused model is trained on preprocessed datasets. In the training process, several hyperparameters are usually initialized using literature knowledge; however, we implemented BO for the selection of hyperparameters. The trained model is later utilized for the testing process. During testing, activation is performed on the fully connected layer, and classification is performed using the softmax classifier. The experimental validation on the ADNI and OASIS datasets showed the strong generalizability of the proposed approach, achieving accuracies of 97.98% and 94.52%, respectively. Furthermore, interpretability through Grad-CAM visualizations highlighted disease-relevant brain regions, bridging the gap between algorithmic decision making and clinical trust. Collectively, these contributions highlight the potential of X-ViTCNN as an effective and interpretable solution for the early and accurate detection of Alzheimer’s disease.

Despite these promising results, several limitations must be acknowledged. Firstly, the fused architecture is computationally intensive, requiring significant GPU resources due to its ~46 M parameters, potentially limiting deployment in low-resource clinical settings. Secondly, the experiments utilized two publicly accessible datasets, which, despite being common benchmarks, may not fully represent broader populations due to variations in imaging protocols and demographic characteristics. Thirdly, reliance on 2D MRI slices rather than volumetric 3D scans could limit the model’s ability to capture the full spatial context, potentially overlooking progression patterns across slices. Additionally, while Grad-CAM provided interpretability, the further integration of advanced explainability methods could improve clinical confidence in stage-specific predictions.

Future work will aim to tackle these constraints. Optimization strategies such as model pruning, quantization, and lightweight transformer variants can be explored to reduce computational overhead while preserving accuracy. Expanding the framework to incorporate 3D volumetric MRI and multimodal data (e.g., PET, fMRI, and clinical biomarkers) could enhance its predictive capacity and clinical applicability. Moreover, advanced explainability techniques, uncertainty estimation, and bias analysis will be integrated to improve transparency and fairness in decision support. Finally, prospective validation in multi-center clinical trials will be essential to assess the reliability, robustness, and integration of X-ViTCNN into real-world healthcare workflows.

## Figures and Tables

**Table 1:** People that participated in ADNI.

Age Group	Female	Male	Unknown
40–49	3	0	0
50–59	101	44	1
60–69	468	372	3
70–79	615	740	0
80–89	191	292	1
Above 89	14	14	4

**Table 2:** ADNI categories and number of images.

Classes	Original	Utilized
CN	7819	2600
EMCI	7290	2600
LMCI	2450	2450
MCI	3871	2600

**Table 3:** Original and utilized class distributions with dataset splits for ADNI and OASIS.

	Classes	Original		Utilized
	Mild Dementia	5002		4666
	Moderate Dementia	488		4670 (Augmented)
	Non-Demented	67,200		4666
	Very Mild Dementia	13,700		4666
	Total	Train	Test	Validation
ADNI	10,250	7175	1537	1538
OASIS	18,668	13,068	2800	2800

**Table 4:** Model parameters and specifications of adopted architecture.

Model	Input Size	No. of Layers	Parameters (Millions)	Core Components	Strengths	Limitations
DenseNet201	224 × 224 × 3	201	~20.2 M	Dense blocks + transition layers	Strong feature reuse, mitigates vanishing gradient	Computationally heavier than lightweight models
MobileNetV2	224 × 224 × 3	53	~3.4 M	Inverted residual blocks, depthwise convolutions	Lightweight, efficient for mobile/embedded	Lower accuracy compared to deeper CNNs
Custom ViT	224 × 224 × 3 (patch size 16 × 16)	12 Encoder Layers	~22.1 M	Multi-head self-attention + MLP	Captures global dependencies, interpretable	Requires large data, training intensive
Proposed Fusion (DenseNet201 + MobileNetV2 + ViT)	224 × 224 × 3	---	~46 M	Depth concatenation + dropout + softmax	High accuracy, combines strengths of CNN and transformer	More parameters, needs GPU resources

**Table 5:** Importance and complexity of adopted architectures.

Model	Architecture Overview	Features	Complexity
DenseNet201	Several dense blocks with direct connections between layers within each block. These connections enable excellent feature reuse and promote gradient flow, thereby mitigating the vanishing gradient issue.	Feature reuse, leveraging dense connectivity patterns to facilitate effective information flow between layers. This approach allows the network to identify complex patterns and relationships within the data.	Intricate
MobileNetV2	Inverted residual blocks with linear bottleneck layers facilitate efficient feature extraction through depthwise separable and expansion convolutions.	Lightweight and efficient architecture, residual connections and linear bottlenecks enhance feature reuse and mitigate the vanishing gradient problem.	Balanced
ViT	Revolutionizes image processing by leveraging transformer-based architecture, initially designed for sequence modeling tasks. It begins by dividing input images into patches, then embedding them into dense representations. Positional encodings are added to provide spatial information, enhancing the model’s understanding of image structure.	Capturing global dependencies and long-range interactions within images through multi-head self-attention mechanisms. It effectively models complex patterns and structures while maintaining interpretability. Additionally, residual connections and layer normalization stabilize training and enhance model robustness.	Moderate

**Table 6:** Results of the classification using the proposed X-ViTCNN architecture with the ADNI dataset.

Models	Accuracy (%)	Precision (%)	Recall (%)	Specificity (%)	F1-Score (%)
Fine-tuned DenseNet201	64.25	78.65	65.08	88.08	63.11
Fine-tuned MobileNetV2	56.5	63.87	57.3	85.49	54.40
Custom ViT	91.33	94.77	91.00	97.67	90.67
Proposed Architecture (X-ViTCNN)	97.98	99.33	97.42	99.67	97.12

**Table 7:** Results of the classification using the proposed fused architecture on the OASIS dataset.

Models	Accuracy (%)	Precision (%)	Recall (%)	Specificity (%)	F1-Score (%)
DenseNet201	67.45	81.22	67.98	91.33	66.43
MobileNetV2	54.02	62.46	55.67	80.46	53.16
Custom ViT	90.88	94.06	89.98	96.33	90.12
Proposed Architecture (X-ViTCNN)	94.52	97.99	96.23	98.16	94.17

**Table 8:** Comparison of the proposed X-ViTCNN model with other architectures on the naturally balanced cohort from the OASIS dataset.

Model	Accuracy (%)	Precision (%)	Recall (%)	Specificity (%)	F1-Score (%)
DenseNet201	67.45	81.22	67.98	91.33	66.43
MobileNetV2	56.50	63.87	57.30	85.49	54.40
Custom ViT	91.33	94.77	91.00	97.67	90.67
Proposed X-ViTCNN	94.52	97.99	96.23	98.16	94.17

**Table 9:** Ablation study on ADNI: the effect of components on the proposed model’s performance. ΔAcc is the absolute accuracy change vs. the full model.

Variant	Accuracy (%)	ΔAcc (pp)	Precision (%)	Recall (%)	Specificity (%)	F1-Score (%)
DenseNet201 (baseline)	64.25	−33.73	78.65	65.08	88.08	63.11
MobileNetV2 (baseline)	56.50	−41.48	63.87	57.30	85.49	54.40
Custom ViT (baseline)	91.33	−6.65	94.77	91.00	97.67	90.67
CNN Fusion Only (DenseNet201 + MobileNetV2, no ViT)	82.40	−15.58	88.12	83.05	92.11	82.70
Element-wise Addition Fusion	90.10	−7.88	94.35	90.25	96.88	89.77
Attention-based Weighting Fusion	93.80	−4.18	96.15	94.80	97.22	94.12
Full Model w/o Contrast Enhancement	95.62	−2.36	96.98	95.02	97.84	95.21
Full Model w/o BO (random/hand-set hparams)	96.34	−1.64	97.45	95.88	98.21	96.05
Full Model (Sum fusion instead of Depth-Concat)	96.02	−1.96	97.20	95.47	98.09	95.74
Full Model (ViT patch size 32, not 16)	95.88	−2.10	96.85	95.42	98.00	95.33
Full Model (X-ViTCNN Fused Network)	97.98	0.00	99.33	97.42	99.67	97.12

**Table 10:** Ablation study on OASIS: the effect of components on the proposed model’s performance. ΔAcc is the absolute accuracy change vs. the full model.

Variant	Accuracy (%)	ΔAcc (pp)	Precision (%)	Recall (%)	Specificity (%)	F1-Score (%)
DenseNet201 (baseline)	67.45	−27.07	81.22	67.98	91.33	66.43
MobileNetV2 (baseline)	54.02	−40.50	62.46	55.67	80.46	53.16
Custom ViT (baseline)	90.88	−3.64	94.06	89.98	96.33	90.12
CNN Fusion Only (DenseNet201 + MobileNetV2, no ViT)	80.56	−13.96	86.34	81.29	91.42	80.77
Full Model w/o Contrast Enhancement	92.85	−1.67	95.07	92.10	96.90	92.30
Full Model w/o BO (random/hand-set hparams)	93.14	−1.38	95.22	92.36	97.05	92.66
Full Model (Sum fusion instead of Depth-Concat)	92.77	−1.75	94.98	92.01	96.74	92.20
Full Model (ViT patch size 32, not 16)	92.41	−2.11	94.65	91.68	96.50	91.82
Full Model (X-ViTCNN Fused Network)	94.52	0.00	97.99	96.23	98.16	94.17

**Table 11:** Comparison of proposed fused architecture with other pre-trained neural networks.

Model	Datasets		Accuracy (%)
	ADNI	OASIS	
Fine-tuned DenseNet201	✓		64.25
		✓	67.45
Fine-tuned MobileNetV2	✓		56.50
		✓	54.02
Fine-tuned ResNet101	✓		51.29
		✓	53.04
Fine-tuned Resnet-50	✓		49.80
		✓	50.26
Inception-Resnet	✓		52.58
		✓	60.04
Proposed Custom ViT	✓		91.33
		✓	90.88
Proposed Fused Network (X-ViTCNN)	✓		97.98
		✓	94.52

**Table 12:** Comparison of existing techniques of AD classification.

Author	Dataset	Method	Accuracy (%)	Precision (%)	Recall (%)	Specificity (%)	F1-Score (%)	XAI
Hazarika et al. [23]	ADNI	Hybrid model using LeNet and AlexNet	93.58	93.33	93.40	-	93.90	No
Hajamohideen et al. [29]	ADNI and OASIS	SCNN with triplet loss function	93.85 with OASIS	93.90	95.61	95.61	94.70	No
Li et al. [43]	ADNI	Efficient Net B2 GCM-EB2	92.60	-	-	-	-	No
Qu et al. [44]	ADNI	UNB-GCN	93.90	98.39	89.13	90.60	94.30	No
Carcagnì et al. [45]	ADNI and OASIS	ResNet, DenseNet, EfficientNet	72.12	-	-	-	-	No
Nagarathna and Kusuma [46]	ADNI	DNN and feed-forward NN (hybrid model)	93.38	89.97	-	95.04	90.00	No
Proposed Model	ADNI and OASIS	Fused network (X-ViTCNN)	97.98 with ADNI	99.33	97.42	99.67	97.12	Yes

**Table 13:** Statistical performance of X-ViTCNN on ADNI and OASIS datasets.

Metric	ADNI (X-ViTCNN)	OASIS (X-ViTCNN)
Accuracy (%)	97.98 ± 0.85	94.52 ± 1.23
95% Confidence Interval	(97.10%, 98.86%)	(93.26%, 95.78%)
Precision (%)	99.33 ± 0.77	97.99 ± 1.15
95% Confidence Interval	(98.47%, 100.19%)	(96.02%, 99.96%)
Recall (%)	97.42 ± 0.91	96.23 ± 1.09
95% Confidence Interval	(96.50%, 98.34%)	(95.12%, 97.34%)
Specificity (%)	99.67 ± 0.72	98.16 ± 0.98
95% Confidence Interval	(98.48%, 100.02%)	(97.23%, 99.09%)
F1-Score (%)	97.12 ± 0.82	94.17 ± 1.12
95% Confidence Interval	(96.29%, 97.95%)	(93.02%, 95.32%)
Paired t-test (*p*-value)	<0.001	<0.01

## Data Availability

The datasets used in the experiment are publicly available, and the URLs are provided below: 1. ADNI Dataset: https://adni.loni.usc.edu/ (accessed on 12 July 2025). 2. OASIS Dataset: https://www.kaggle.com/datasets/ninadaithal/imagesoasis (accessed on 12 July 2025).
